# Large Area Nanohole Arrays for Sensing Fabricated by Interference Lithography

**DOI:** 10.3390/s19092182

**Published:** 2019-05-11

**Authors:** Chiara Valsecchi, Luis Enrique Gomez Armas, Jacson Weber de Menezes

**Affiliations:** Engineering Department, Universidade Federal do Pampa, Alegrete 97546-550, RS, Brazil; luisarmas@unipampa.edu.br

**Keywords:** holography, optics at surfaces, surface plasmons, subwavelength structures, nanostructures, biological sensing and sensors

## Abstract

Several fabrication techniques are recently used to produce a nanopattern for sensing, as focused ion beam milling (FIB), e-beam lithography (EBL), nanoimprinting, and soft lithography. Here, interference lithography is explored for the fabrication of large area nanohole arrays in metal films as an efficient, flexible, and scalable production method. The transmission spectra in air of the 1 cm^2^ substrate were evaluated to study the substrate behavior when hole-size, periodicity, and film thickness are varied, in order to elucidate the best sample for the most effective sensing performance. The efficiency of the nanohole array was tested for bulk sensing and compared with other platforms found in the literature. The sensitivity of ~1000 nm/RIU, achieved with an array periodicity in the visible range, exceeds near infrared (NIR) performances previously reported, and demonstrates that interference lithography is one of the best alternative to other expensive and time-consuming nanofabrication methods.

## 1. Introduction

Since the discovery of Ebbesen on the extraordinary optical transmittance (EOT), many works have been carried out to explore its potential in sensing applications [1,2,3,4,5,6,7,8,9,10,11]. Most of the theoretical works attribute EOT to the excitation of surface plasmons (SP) at metal-dielectric interfaces [1,2,3,4,5,6,7]. SPs are surface bound electromagnetic waves that can be excited by free-space radiation using periodic nanostructures at metal surfaces [1,2,3,4,5,6,7], and the variation in the dielectric constant at the gold-dielectric interface of the substrate produces a shift in the transmitted light peak positions [1,2,3,4,5,6,7]. In the simplest approximation, an SP mode can be excited when the wave vectors of the incident light and periodic structure match the conditions for momentum conservation. When the periodic structure is an array of nanoholes, the SP resonance leads to an enhancement in the transmitted light. For normal incidence in a squared array of holes, the wavelength position of such transmittance peaks is given by:

(1) $$\lambda_{0}=\frac{\Lambda }{\sqrt{i^{2}+j^{2}}}{\left(\frac{\varepsilon_{M}\varepsilon_{D}}{\varepsilon_{M}+\varepsilon_{D}}\right)}^{1/2},$$

with Λ being the period of the array, *ε_D_* the dielectric constant of the surrounding medium and *ε_M_* the dielectric constant of the metal. Each pair of integers *i* and *j* represents a SP mode excited at the metal interface. It is common to have different dielectrics on each side of the metal film: the substrate that supports the metal film *ε_s_* and the upper medium in direct contact to the metal surface *ε_u_*. Thus, two different kinds of transmittance peaks are expected: due to the metal–upper surrounding medium interface, and due to the metal–supporting substrate, for each SP mode (*i*, *j*). A more detailed investigation shows that the resonances on either side of the metal film are actually coupled through the nanoholes, and this will shift the peak positions relative to the predicted by Equation (1) [5,6].

As a natural consequence, the use of the spectral shifts to detect surface binding has been demonstrated [1,2,3,4,5,6,7], and indeed, the nanohole array system has been the most explored substrate as a highly-integrated and multiplexed surface plasmon resonance (SPR) platform [3,4,5,6,7,10,11]. 

Among several aspects, as the optics for resonance excitation, signal detection, and sensing method (microfluidic, flow through, as examples), the fabrication of nanoholes in gold films is one of the most critical aspects of the whole sensing performance [4,5,6,7]. The substrate reproducibility and the exact control of geometrical parameters, such as hole diameter, periodicity, film thickness, and structure ordering, assume key roles in the final measurement sensitivity [11,12,13]. Moreover, other prime factors to consider when fabricating nanohole arrays are costs, manufacturing speed and wide-scale deployment of the nanofabrication technique used [14].

In the early years, nanoholes have been fabricated mainly through focused ion beam (FIB) milling or electron beam lithography (EBL) [1,2,3,4,5,8,9,10,11,12]. These techniques allow the precise fabrication of highly-ordered arrays with a well-defined geometry and well-controlled hole-size. Moreover, the bulk sensitivity of pure nanohole arrays, normally used as a benchmark for comparisons, is found in the order of 400–650 nm/RIU in the visible range [3,4,5,15]. 

However, the serial nature of FIB and EBL milling means a slow production, and the elevated costs cause that typical arrays are less than 100 μm × 100 μm. To obtain highly-multiplexed sensing, eventually integrated with microfluidics, a much larger array field is desired, in order to address several sensing areas. For this reason, a parallel and fast fabrication method becomes necessary. In order to suppress these limitations, in the past decades several different techniques have been explored and evaluated for the production of nanohole arrays: UV nanoimprinting [16,17,18,19], nanosphere lithography [20,21], soft lithography [22,23,24], and interference lithography [25,26,27] are, among others, the most common examples.

UV nanoimprinting is a pattern transfer technique, in which a unique mold, mostly fabricated by electron beam, is replicated onto a photoresist several times [16,17]. Although a high flexibility in terms of geometrical parameters is achieved, depending on the mask used, the resolution of the mold and the cost associated with it are two important limiting factors, together with the relatively low bulk sensor sensitivity demonstrated (from 150 nm/RIU up to 316 nm/RIU in the visible) [18,19]. Overall, this method of fabrication has gained more attention for the production of localized surface plasmon substrates (LSP), as nanodisks and nanoparticles [28,29].

Nanosphere lithography (NSL), also known as colloidal lithography or natural lithography [30], is another fabrication method introduced for the fabrication of LSP sensors by the work of Van Dyne and colleagues [31,32], which was further explored in several works as a self-assembly method for nanoholes array fabrication [20,21]. In this technique, the nanometric particles (mostly polystyrene) naturally tend to agglomerate into a regular hexagonal pattern, covering uniformly large areas of the substrate. However, after deposition, the substrate has to be submitted to a plasma or a reactive ion etching (RIE) step, in order to decrease the sphere diameter and to create sufficient surface access for the metal deposition and final sphere removal. The bulk sensitivity achieved with this substrate in the NIR, at 1000 nm, was found to be in the same order of the sample fabricated by FIB, around 487 ± 51 nm/RIU [21]. Therefore, large areas can be fabricated with little spatial defects (although particle agglomeration and domain-like defects might occur) and relatively good performance. On the other hand, this fabrication method requires different steps and techniques that increase the overall costs and complexity of the application for a mass production system.

Wu et al. [33,34] introduced a different approach in order to diminish the complexity of the NSL fabrication by using pure laser lithography associated with the concept of spheres acting as optical lenses: although this method can greatly contribute to the production of nanoscale patterns, including nanohole arrays [33,34,35], to our knowledge, no nanohole sensing platforms have been tested with this method.

Soft lithography and soft interference lithography also gained interest as a nanopattern fabrication technique, being the first introduced by Whiteside [22] and the latter, more recent, by Odom and collaborators [23]. The fabrication exploits the versatility of polymers, mostly polydimethylsiloxane (PDMS), as replicating photolithography masks. The mask is produced once by replicating a silicon wafer patterned by EBL, and it can be reused several times to replicate the nanohole array onto a new substrate. Another central step is the peeling or stripping process, which improves the gold surface roughness, diminishing propagation losses, for a better sensitivity [7]. Henzie et al. registered a bulk sensitivity of ~300 nm/RIU [23], while Jiu et al. reported a 522 nm/RIU sensitivity [24], both for the 770 nm resonance peak. On the bright side, soft interference lithography is a cheap, versatile, and high-throughput technique, limited only by the deformation of the flexible PDMS mask. On the other side, the actual transfer of the photoresist pattern onto metal films, called PEEL (a combination of phase-shifting photolithography, etching, electron-beam deposition, and film lift-off) [23], requires the use of several techniques, limiting the sensor mass production.

By analyzing the pros and cons of all the techniques cited above, pure laser interference lithography still persists as a very interesting alternative for the generation of high-resolution periodic structures on large areas [25,26,27]. By using a double exposure of a photoresist film to the same interference pattern, large area two-dimensional arrays can be recorded with great geometry tailoring, reproducibility, high speed, low cost, and high sensor performance [25,26,27]. No mask or pre-treatment are required, the hole diameter can be tuned during the developing step, and depending on the laser and photoresist used, the hole periodicity can be tuned in a wide range, as desired. With a ready-to-go setup, the output might be as high as 10 substrates per hour (considering the metal evaporation step). 

In this paper, we therefore demonstrate the optimum control on the geometrical aspects and the great versatility of the arrays produced by interference lithography, presenting the effects of periodicity, hole-diameter and film thickness on the surface plasmon peaks. Additionally, the performance as a bulk plasmonic sensor was evaluated in light of the previous analyses and a small comparison with platforms fabricated by different techniques was added. The results demonstrate that interference lithography is a cost effective, flexible, and efficient technique for the fabrication of nanopattern for sensing. 

## 2. Nanohole Array Fabrication

**Figure 1:**
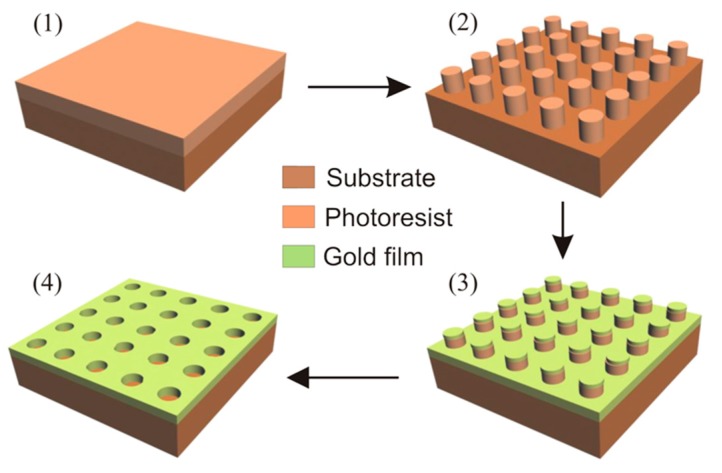
Scheme of nanohole array fabrication. (1) Photoresist deposition by spin coating, (2) double exposure of two-beam interference patterns using λ = 458 nm followed by photoresist development. (3) Au film deposition by thermal evaporation and (4) lift-off of the photoresist template.

Figure 1 shows a schematic of the experimental procedure employed for recording the metallic arrays. In the first step, the positive photoresist (SC 1827 Rohm and Haas) was deposited by spin coating on glass substrates previously covered with a thin film of the negative photoresist SU-8 (from Microchem, Westborough, MA, USA) in order to improve the adhesion. The SC 1827 was spin coated using a rotation of 3500 rpm for 30 s. These conditions lead to a film thickness of about 550 nm. After the pre-bake at 70 °C during 20 min., the photoresist was exposed twice to the same interference pattern, by rotating the sample of 90° between the exposures. Although the fabrication here presented is for a square lattice geometry, it is possible to generate other patterns depending on the substrate rotation and number of exposures [36]. The interference pattern is provided by an interferometric setup using a 300 mW Melles Griot solid-state laser operating at λ = 458 nm. The interferometer in this work is constituted by the superimposition of two beams and the schematics and details about the optical setup is available in [37]. Our setup allows the recording of fringe periods from 450 nm to 1800 nm and it is provided with a fringe locker system, which warrants the high contrast of the fringe pattern and the repeatability of the exposures [37]. Samples with periods of Λ = 575 nm and Λ = 675 nm were fabricated for this work. After the double exposure at the same dose of 400 mJ/cm^2^, the photoresist film was developed in AZ Developer diluted 1:4 in order to obtain the photoresist template. These conditions allow to generate structures with high aspect ratio. The development time was varied in order to achieve the desired hole diameter. The last step is the thermal evaporation of a thin Au film, followed by the lift-off process of the photoresist template in acetone. Different film thicknesses have been deposited on the samples considering a gold deposition rate of 1.6 nm/mg.

## 3. Results

**Figure 2:**
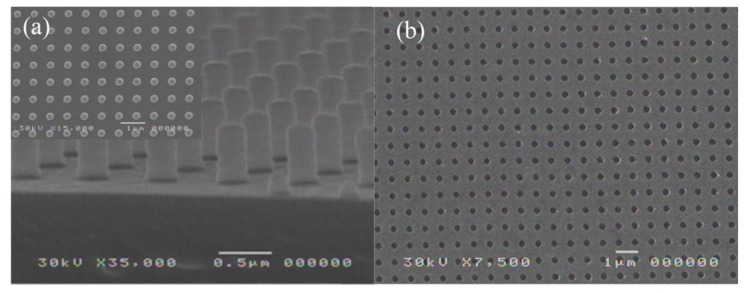
Scanning Electron Microscopy (SEM) photograph of the photoresist template on the glass substrate (**a**). The inset shows the top-view of the template. (**b**) Top-view of the array of holes recorded in the Au film after the lift-off of the photoresist. Periodicity: 675 nm; diameter: 280 nm; gold thickness: 130 nm.

An example of fabricated substrate is displayed in Figure 2. Particularly, Figure 2a shows two Scanning Electron Microscopy (SEM) images of the cross-section and top-view (inset) of the photoresist template, with a 675 nm period. The photoresist template is constituted of well-defined columns, 550 nm in height, and 280 nm in diameter. This high aspect ratio is a necessary condition for the lift-off process (allowing thickness up to half of the periodicity). Figure 2b shows the SEM photograph of the resulting 2D squared arrays of holes, after the lift-off of the photoresist template using acetone, for an Au film thickness of 130 nm. The samples present a hole diameter dispersion of less than 10% over a square inch. A short movie representing the uniformity of the nanoholes over the whole substrate surface is available in the Supplementary Information.

Primarily, the nanohole arrays with different geometrical parameters fabricated by interference lithography were evaluated by analyzing the transmission spectra in air. The spectra were compared, when possible, with the expected theoretical values of the plasmon resonance peaks (Equation (1)) and they were studied in order to verify the effects of different thicknesses, hole diameters and periodicities on the position of the plasmon peaks to optimize the sensing platform.

Firstly, a correlation on the effect of different substrate periodicity was carried out and compared with the theoretical values, and then the influence of different thickness of the gold film on the transmitted peaks was evaluated. At last, maintaining the same thickness and period, the effect of different diameters on the resonance peak position was recorded and examined.

The transmission spectra of the arrays of holes were measured using the λ-9 Perkin Elmer spectrophotometer (UV-VIS-NIR). Because of the large area of the samples (1 square inch), the light beam impinged over the whole nanohole fabricated area. For this reason, they can be placed directly in the sample holder (cuvette) of the spectrophotometer without any additional optics. 

**Figure 3:**
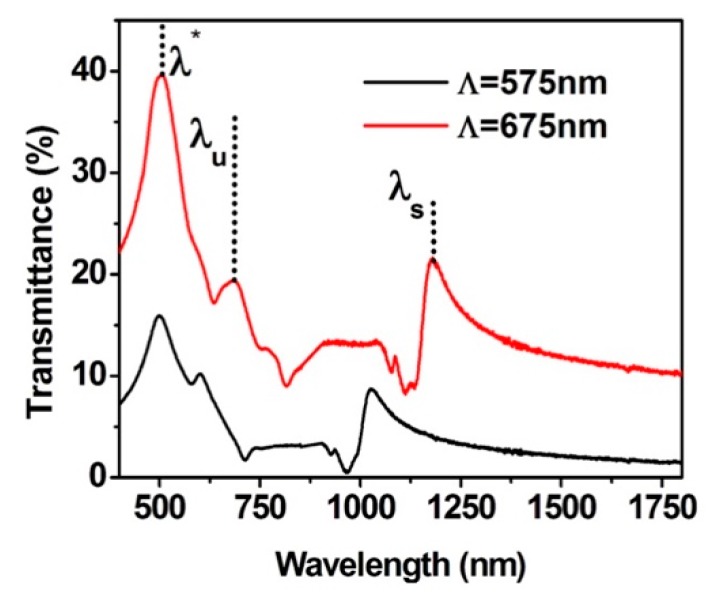
Optical transmission spectra of the array of holes recorded in Au film with 60 nm of thickness, periods Λ = 575 nm (black) and 675 nm (red) and diameter of 230 nm and 250 nm, respectively. The red curve is off-set along the vertical axis in order to separate the curves for comparison purpose.

Figure 3 shows the normal incidence transmission spectra through arrays of holes with 60 nm-thick Au films, fabricated with different periodicity, 575 nm and 675 nm, and very similar hole diameters, 230 nm and 250 nm, respectively. In this situation, it was possible to verify the variation of the surface plasmon resonance peak in respect to the variation of hole periodicity. In the same figure, the well-defined peak at the same wavelength $\lambda^{*}$ = 505 nm (in both curves) corresponds to the combination of interband absorption with the free carrier response [38]. Another small peak, marked as $\lambda_{u}$, is highlighted adjacent to $\lambda^{*}$, together with a second peak, $\lambda_{s}$, at longer wavelengths. As predicted, these two transmittance peaks are due to the metal—upper surrounding medium interface ($\lambda_{u}$), and due to the metal—supporting substrate ($\lambda_{s}$). The position of both $\lambda_{u}$ and $\lambda_{s}$ depends on the periodicity of the arrays and it can be estimated by the SPR analytical equation, Equation (1). The wavelength positions of both $\lambda_{u}$ and $\lambda_{s}$ are summarized in Table 1 in the discussion section.

**Figure 4:**
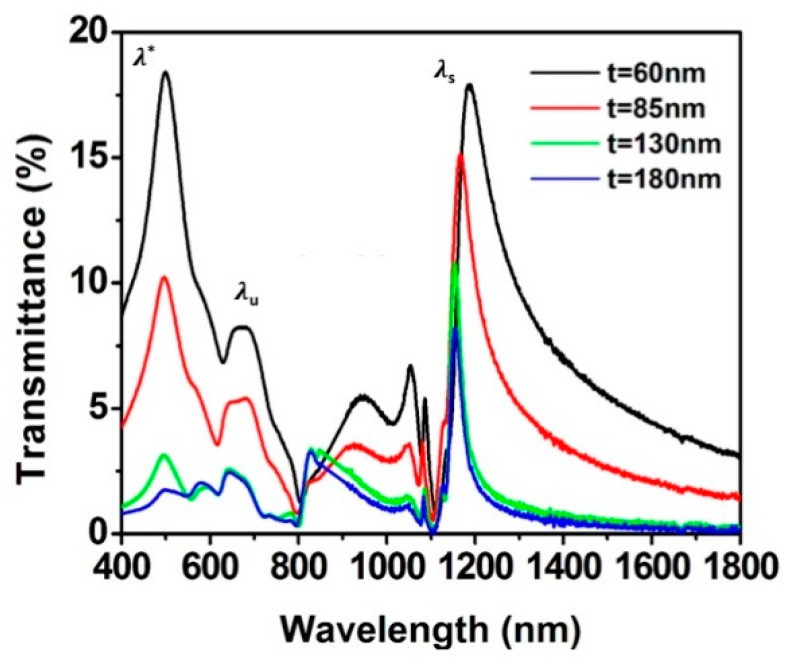
Transmission spectra (in air) of different samples with period of 675 nm, diameter of 280 nm and Au film thickness of 60 nm (black), 85 nm (red), 130 nm (green), and 180 nm (blue).

Figure 4 displays the transmission spectra of samples with the same period (Λ = 675 nm) and hole diameter (D = 280 nm), but different Au film thicknesses (t = 60, 85, 130, and 180 nm). Again, the experimental SPR wavelength positions of both $\lambda_{u}$ and $\lambda_{s}$ resonance peak, as defined previously, were recorded for each film thickness and they are summarized in Table 1. 

**Figure 5:**
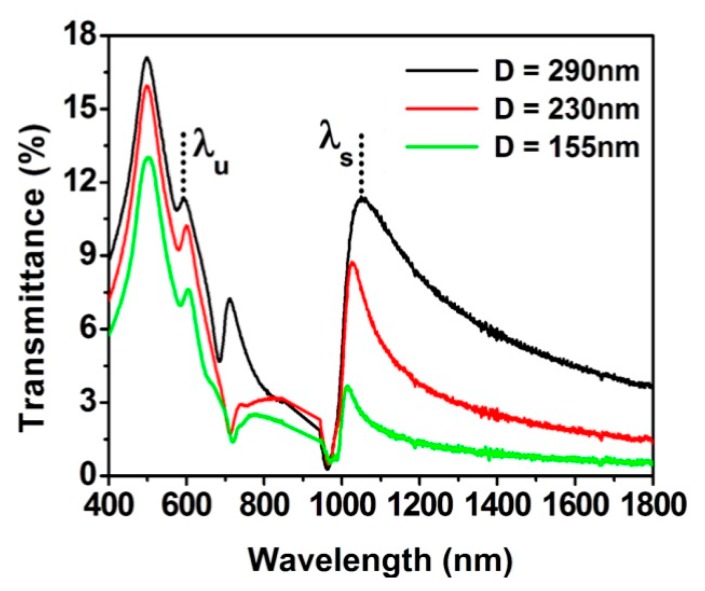
Transmission spectra (in air) of samples with period of 575 nm and Au film thickness of 60 nm, for different hole diameters: 290 nm (black), 230 nm (red), and 155 nm (green).

Figure 5 shows the optical spectra of samples with the same period (Λ = 575 nm) and film thickness (t = 60 nm), but different hole diameters (D = 290, 230, and 155 nm). The wavelength positions of both $\lambda_{u}$ and $\lambda_{s}$, highlighted in the figure, are also reported in Table 1. 

In order to check the applicability of these arrays for refractive index sensing, the zero-order transmission spectra through the squared array of nanoholes were measured for all the sample geometries introduced above, immersed in three different surrounding media: water (n = 1.3280), glycerin (n = 1.4700) and a mixture of water/glycerin (1:1 in volume, n = 1.4078). To perform the measurements in different refractive index media, the samples were placed in the spectrophotometer chamber inside a cuvette filled with the different liquids. After each measurement, the samples were washed with DI water and dried with a nitrogen flow.

**Figure 6:**
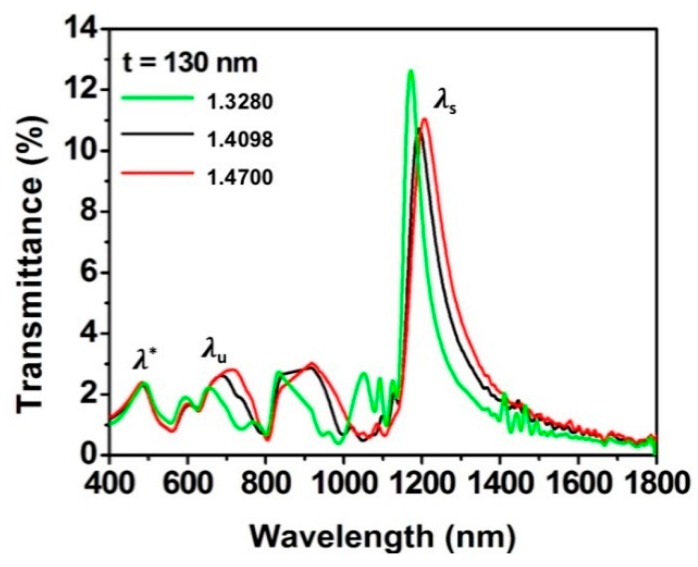
Transmission spectra of the sample with period of 675 nm, Au film thickness of 130 nm and hole diameters of 280 nm in different surrounding media.

As an example, the transmission spectra for the sample with Λ = 675 nm, t = 130 nm, and D = 280 nm immersed in these three different media are shown in Figure 6.

For all the bulk sensing cases, the well-defined peak around 500 nm found in air, due to the combination of interband absorption with the free carrier response, here is much weaker. Also, the resonance peak due to the metal—upper surrounding medium interface, $\lambda_{u}$, can be identify in the spectra, even if not as salient as before. On the other hand, the peak due to the metal—supporting substrate, $\lambda_{s}$, is still intense and sharp. 

**Figure 7:**
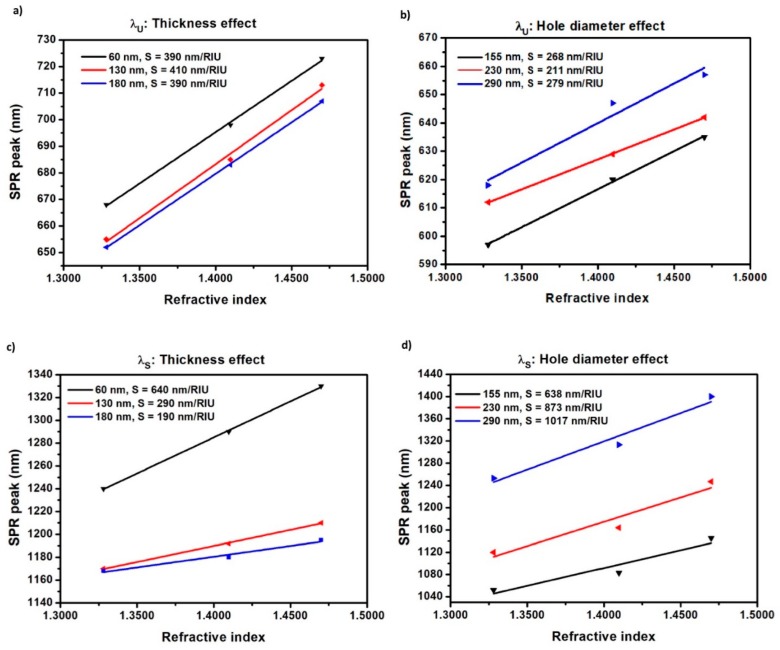
SPR peak position corresponding to $\lambda_{u}$ and $\lambda_{s}$ as a function of refractive index for different film thickness and diameters of the fabricated nanohole arrays (**a**) sensitivity variation for $\lambda_{u}$ in samples with different thicknesses (Λ: 675 nm, D: 280 nm); (**b**) sensitivity variation for $\lambda_{u}$ in samples with different diameters (Λ: 575 nm, t: 60 nm); (**c**) sensitivity variation for $\lambda_{s}$ in samples with different thicknesses (Λ: 675 nm, D: 280 nm), and (**d**) sensitivity variation for $\lambda_{s}$ in samples with different diameters (Λ: 575nm, t: 60 nm).

The bulk sensitivity (*S*) of the substrate was defined as the slope of the calibration curve S=Δλ/Δn, where Δλ is the wavelength shift of the SPR peak ($\lambda_{u}$ or $\lambda_{s}$) and Δn is the refractive index difference of the medium. The achieved sensitivity in respect to the bulk refractive index changes are summarized in Figure 7 for the different samples used. 

Particularly, in Figure 7a and b the sensitivity obtained by monitoring the shift of the $\lambda_{u}$ peak are shown, first for samples with increasing thickness (period and diameter constant, 675 nm and 280 nm, respectively), and second for substrates with increasing hole diameter (period and thickness constant, 575 nm and 60 nm, respectively). The same analysis of sensitivity, although for the $\lambda_{s}$ peak, are represented in Figure 7c,d.

Overall, it can be observed from Figure 7 that the sensitivity (S) of the $\lambda_{s}$ peak increases more than three times and almost doubled when the Au film thickness decreases and the hole-diameter increases, respectively. On the other hand, the increasing thickness and hole diameter have a much less influence on the sensitivity of the $\lambda_{u}$ resonant peak.

The best sensing performance of 1017 nm/RIU was found for the nanohole array with 290 nm diameter, 575 nm periodicity and 60 nm thickness, presenting a resonant peak in the NIR region, around 1050 nm, as shown in Figure 6.

## 4. Discussion

All the surface plasmon resonance peaks $\lambda_{u}$ and $\lambda_{s}$ recorded in air, from Figure 3, Figure 4 and Figure 5 above, are summarized in Table 1. 

As the Au film is surrounded by air and substrate (SU-8), from Equation (1) and for Λ = 575 nm, the two peaks $\lambda_{u}$ and $\lambda_{s}$ for the first SP mode (±1,0) are expected to be in the visible range ($\lambda_{u}$ ~ 608 nm) and in the IR (at $\lambda_{s}$ ~ 1077 nm). In the case of Λ = 675 nm, the expected values from Equation (1) for $\lambda_{u}$ and $\lambda_{s}$ are ~700 nm and ~1190 nm, respectively. The optical constants for gold were taken from Johnson and Christy [39], while the refractive index of SU-8 was considered a constant average value of 1.600 from [40]. These wavelength values agree very well with the positions of the experimental peaks shown in Figure 3 and reported in the first section of Table 1. The small difference can be appointed to the fact that Equation (1) does not consider other geometrical parameters, like hole diameter and gold film thickness. From Figure 3, it is possible to note that, as expected, the peaks ($\lambda_{u}$ and $\lambda_{s}$) red shift when the period of the array increases from 575 to 675 nm [3,4,5,6,41]. Moreover, the experimental spectra of Figure 3 shows that the longest wavelength EOT peak $\lambda_{s}$, corresponding to the gold-substrate interface, is more intense when it is compared with the upper interface peak, $\lambda_{u}$. This fact can be justified as the lower resonance orders of the upper interface can be convoluted with the higher orders of the bottom interface, corrupting the signal [6,41]. For example, considering the case of a 675 nm sample periodicity, gold refractive index of 0.13767, metal extinction coefficient of 3.7917 (calculated using ref [40] and the wavelength of 670 nm), for the gold-air interface and resonance order of (1,0), the resonance peak should be ~700 nm (λ_u_ in Figure 3); on the other hand, for the same conditions, for the gold-substrate interface but order (1,1), the peak would be ~765 nm (little shoulder in Figure 3). The convolution of these bands leads to broader signals, being more difficult to measure changes for sensitivity evaluation. This suggests that the bottom interface can be used as a refractive index sensor once the first order plasmon peak is well-isolated. 

From Figure 4, it is possible to perceive that the bandwidth and the intensity of the $\lambda_{s}$ peak are very sensitive to the thickness, and a narrowing is observed as the film thickness increases from 60 nm to 180 nm. Furthermore, there is a significant reduction, almost 50%, in the amplitude of the spectra for both $\lambda_{u}$ and $\lambda_{s}$ peaks and a slight blue-shift of the wavelength positions as the film thickness increases. This might be appointed to the weaker coupling of the SPR on the two sides of the film and a longer resonance lifetime for thicker layers [4], both of these effects were also found and reported by Wu et al. in [42] and Meunier et al. in [4]. 

From Figure 5 and the last section of Table 1, it is possible to note that, as the hole diameter increases, the overall transmittance increases and the position of $\lambda_{s}$ red-shifts On the other hand, the $\lambda_{u}$ peak experiences a small blue shift. The increase in transmittance and the red-shift of the $\lambda_{s}$ peak with the hole-diameter has been previously reported [4,13,41] and it can be associated with a larger cut-off frequency [4]. This effect indicates that a larger hole might favor the sensing capabilities of the nanohole arrays, as it is easier to differentiate the signal from the transmitted peak with respect to noise or to detect other small intensity higher-order peaks.

From Figure 6, a red shift in the wavelength position of both $\lambda_{u}$ and $\lambda_{s}$ peaks was observed as the refractive index of the surrounding media increases. This effect is well-known in the literature, as reported in [3,4,5,6,7,8,9]. The dependency of $\lambda_{s}$ with the surrounding media can be explained by considering the coupling between the SP modes at the two sides of the film [6]. The two resonances ($\lambda_{u}$ and $\lambda_{s}$) from each side of the Au film are coupled, like coupled oscillators. If the resonances are degenerate, there is a peak splitting for the in-phase and out-of-phase coupling conditions [43]. The peak splitting increases as the coupling is made stronger. Therefore, if $\lambda_{u}$ changes with the surrounding media, the $\lambda_{s}$ peak also changes due to coupling [6,43]. Therefore, it is expected that the stronger the coupling, the greater the effect of the refractive index on the upper side of the film, which translates to larger peak shift and larger sensitivity. Following this reasoning, two aspects can be seen in Figure 7: first, the larger sensitivities to refractive index changes were indeed observed from the samples with thinner film and wider hole diameter. Particularly, for a 575 nm periodicity, the best sensing performance of 1017 nm/RIU was found for the substrate with 290 nm hole diameter and 60 nm gold film thickness. Secondly, the $\lambda_{s}$ peak in Figure 7c and d presented higher sensitivity to refractive index changes than $\lambda_{u}$ (Figure 7a,b) for almost all the cases studied, except for 130 nm and 180 nm thick films with constant diameter (Figure 7a). The difference between the two resonance peaks $\lambda_{u}$ and $\lambda_{s}$ was up to 3.6 times for the best sensing conditions. Most importantly, the sensitivity of the $\lambda_{u}$ plasmonic peak was only slightly affected by the changes in thickness, or the changes in hole aperture, as it was already reported by Hajiaboli et al. [44].

At last, it is worth to compare the performance obtained by this sensor platform with substrates from other fabrication techniques comparables in terms of costs, simplicity, and mass production applicability, as cited earlier in this paper. Remembering that our periodicity is in the order of the visible light (575 or 675 nm), when looking at the $\lambda_{u}$ resonance in the visible range, the performance encountered was found in the same order of magnitude (~400 nm/RIU) of other achievements presented in several works with platforms fabricated by FIB, e-beam, nanosphere, and soft lithography [3,4,5,18,19,21,23,24]. On the other hand, the value of ~1020 nm/RIU obtained in our work for $\lambda_{s}$, without any additional data treatment, exceed the sensitivities in the NIR found in the literature for other fabrication techniques for the same resonance peak. Particularly, Sharpe et al. achieved a sensitivity of 393 nm/RIU with arrays fabricated by e-beam, while Yanik et al. reported a 630 nm/RIU performance on a free-standing array by e-beam and RIE [45,46]; Murray-Methot et al. obtained a sensitivity of 487 ± 51 nm/RIU with a substrate fabricated with NSL [21] and Soler et al. demonstrated a sensitivity of 600 nm/RIU on a free-standing gold nanohole array fabricated by deep UV lithography [47]. Therefore, the results achieved allow to demonstrate the large flexibility of sample fabrication by interference lithography and the great applicability of this technique for sensing platform production.

## 5. Conclusions

With this work, it was demonstrated the flexibility of the interference lithography technique to fabricate nanohole pattern substrates with tailored metal film thickness, due to the structure high aspect ratio, periodicity, and hole size. Most importantly, the possibility to produce different combinations of geometrical array parameters allows to tune the shape and location of the resonance transmission peak. Particularly, the periodicity defines the position of the $\lambda_{u}$ and $\lambda_{s}$ peak: at longer wavelength (NIR) the resonance peak is well-defined and isolated, providing better conditions to measure spectral shifts. The thickness of the gold layer and the diameter of the holes have a fine tune influence on the peak position and affect the peak sharpness and intensity. Particularly, the thickness was found to influence the shape and the intensity of the $\lambda_{s}$ peak, while a larger diameter can be associated with an increased coupling to the substrate SPR resonance through the nanoholes and, therefore, a more intense transmission. The highest bulk sensitivity of ~1020 nm/RIU was found to be superior to other fabrication methods presenting similar geometric parameters of the sample and experimental optical measurements (zero-order transmission spectra; NIR resonance peak; no post-processing treatment of data). Finally, the interference lithography approach provides mass fabrication of broad-area arrays with low dispersion, at low cost and with high throughput. The broad-area arrays are of particular interest for future investigations of highly-multiplexed sensors.

## Figures and Tables

**Table 1:** Data from experimental transmission spectra for arrays of holes in air for different periods, film thickness and hole diameter. Λ: periodicity; T: thickness; D: diameter; $\lambda_{u}$: upper-metal resonance peak; $\lambda_{s}$: metal-substrate resonance peak.

Λ (nm)	T (nm)	D (nm)	λ_u_	λ_s_
			(nm)	(nm)
575	60	230	601	1027
675	60	250	670	1178
675	60	280	679	1186
675	85	280	662	1166
675	130	280	645	1154
675	180	280	645	1154
575	60	155	605	1015
575	60	230	601	1027
575	60	290	594	1050

## Supplementary Materials

The following are available online at https://www.mdpi.com/1424-8220/19/9/2182/s1, Video S1: Uniformity of large area (1 inch squared) nanoholes fabricated by Interference Lithography. Part of the sample, about 1 cm, is moved inside the SEM microscope and the live micrographs are filmed.
